# The Antitumor Potential of Celastrol: Research Progress on Antitumor Mechanisms and Strategies for Toxicity Reduction with Efficacy Enhancement

**DOI:** 10.3390/biom16050620

**Published:** 2026-04-22

**Authors:** Qian Jiang, Zhaojing Liu, Jie Cheng, Zhiyuan Geng, Yu Gu, Yan Wei, Mengjia Yu, Yi Bi

**Affiliations:** School of Pharmacy, Key Laboratory of Molecular Pharmacology and Drug Evaluation (Yantai University), Ministry of Education, Collaborative Innovation Center of Advanced Drug Delivery System and Biotech Drugs in Universities of Shandong, Yantai University, Yantai 264005, China; 202300362011@s.ytu.edu.cn (Q.J.); 202400362081@s.ytu.edu.cn (Z.L.); ytucj666@s.ytu.edu.cn (J.C.); gzy15224366434@s.ytu.edu.cn (Z.G.); 202400362064@s.ytu.edu.cn (Y.G.); 202300362089@s.ytu.edu.cn (Y.W.); 202362501239@s.ytu.edu.cn (M.Y.)

**Keywords:** celastrol, antitumor mechanism, toxicity, derivatives, dosage form optimization

## Abstract

Celastrol, a natural compound with potent antitumor activity, has gained considerable attention in drug research and development. Although its use is limited by high toxicity, a narrow therapeutic window, and severe side effects, studies show that celastrol can inhibit tumors through multiple targets and pathways, including by inducing apoptosis, autophagy, as well as suppressing invasion and adhesion. It also enhances antitumor effects by reshaping the immunosuppressive tumor microenvironment, regulating stromal components, and suppressing angiogenesis. This review systematically summarizes recent advances in related research and elaborates on the molecular mechanisms underlying the antitumor activity of celastrol and the key factors contributing to its toxicity. In addition, we further discuss current progress in research focused on reducing celastrol’s toxicity and enhancing its efficacy, aiming to promote its safety and druggability through rational structural modification, target optimization, and advanced formulation development.

## 1. Introduction

Malignant tumors represent a serious threat to human health. According to recent reports, the global incidence of malignant neoplasms is expected to exceed 35.3 million cases by 2050, representing a 76.6% increase from the 20 million cases recorded in 2022. Furthermore, it is estimated that 18.5 million people will die from malignant tumors by 2050. In low-income countries, the number of cases and deaths is expected to nearly triple [1]. Currently, a variety of antitumor drugs have been approved worldwide, but their use remains limited by drug-induced toxicity and tumor multidrug resistance. Therefore, the search for more effective antitumor drugs is urgent [2].

**Figure 1:**
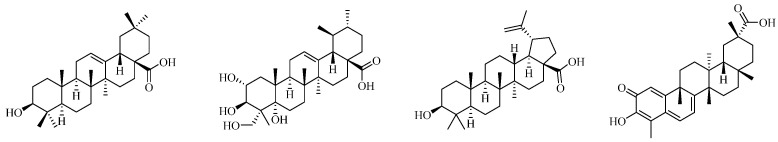
Structures of representative pentacyclic triterpenoids.

Natural products and their derivatives have demonstrated significant potential in antitumor research due to their unique chemical structures and multi-target mechanisms. Due to their structural diversity and multiple pharmacological activities, such as antitumor, anti-inflammatory, hepatoprotective and antidiabetic effects, pentacyclic triterpenoids have become a key focus in natural product chemistry and novel drug development [3]. As early as 2007, the journal *Cell* identified celastrol, artemisinin, triptolide, capsaicin, and curcumin as natural active compounds with the greatest potential for development into modern medicines [4]. These compounds are characterized by a five-membered ring skeleton, with major structural types including ursane (Asiatic acid), oleanane (Oleanolic acid), lupane (Betulinic acid), and friedelane (Celastrol), as illustrated in Figure 1.

Celastrol is a pentacyclic triterpenoid natural product isolated from *Tripterygium wilfordii Hook. f.*, a plant of the Celastraceae family. It has a molecular formula of C_29_H_38_O_4_ and a melting point of 185–200 °C. Celastrol appears as a red crystalline powder and is soluble in solvents such as dimethyl sulfoxide, ethanol, and dimethylformamide, but insoluble in water. Owing to its unique biological activity, celastrol exerts antitumor effects through multiple targets and pathways in various cancer cells, such as hepatocellular carcinoma [5], lung cancer [6], ovarian cancer [7], breast cancer [8], and gastric cancer [9].

Despite the considerable attention that celastrol has received in pharmaceutical research due to its diverse biological activities, including anti-inflammatory and antitumor effects, its clinical translation in cancer therapy remains at an early stage [10]. This limitation is primarily due to its inherent high toxicity, which includes hepatotoxicity, nephrotoxicity, hematopoietic toxicity, and cardiotoxicity, posing serious risks to patient health [11]. These toxicities are major obstacles to its clinical application. In addition, celastrol has extremely poor solubility, with an oral bioavailability of only 17.06% [12]. Its dissolution and absorption in vivo are significantly limited, which restricts its therapeutic efficacy. The C-3 hydroxyl group, the A/B ring, and the C-20 carboxyl group of celastrol can all be modified to address high toxicity, poor water solubility, and low bioavailability. This provides multiple opportunities for developing celastrol derivatives. To overcome its severe toxic side effects, current studies have focused on three main strategies: identification of toxic targets, molecular structural modification, and formulation optimization.

**Figure 2:**
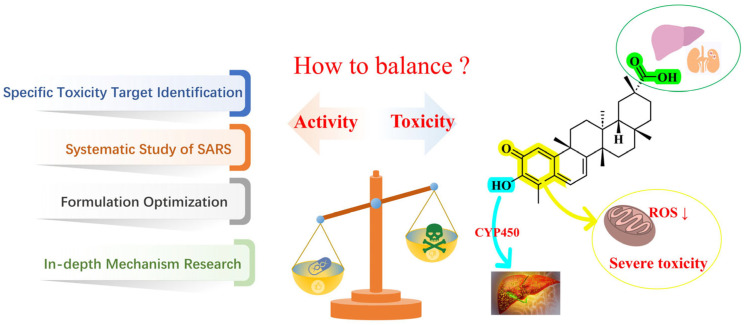
Balancing activity and toxicity of celastrol.

The innovation of this review lies in providing a systematic overview and integration of existing studies by comprehensively summarizing the broad-spectrum antitumor mechanisms of celastrol, the molecular structural basis of its toxicity, and strategies for toxicity reduction. It also elucidates the balanced regulatory relationship between its antitumor activity and toxicity, as illustrated in Figure 2. This review focuses on the core issue of high toxicity, analyzes its underlying causes, and systematically presents corresponding solutions. Beyond classical cell death mechanisms, it further discusses the dual roles of apoptosis and highlights recent advances in novel cell death pathways, including ferroptosis, cuproptosis, and pyroptosis, which contribute to the antitumor activity of celastrol, thereby enriching the understanding of its mechanistic network. In addition, the review supplements and refines the comprehensive regulatory network of celastrol in the tumor microenvironment, further extending the depth and breadth of celastrol-related antitumor research. Overall, it provides a theoretical foundation for future mechanistic investigations and strategies aimed at reducing the toxicity of celastrol.

## 2. Mechanisms of the Antitumor Activity of Celastrol

### 2.1. Multidimensional Regulation of Tumor Cell Death

#### 2.1.1. Cell Cycle Arrest

The cell cycle is a highly regulated process that coordinates various cellular mechanisms essential for life to ensure uninterrupted cell division and the precise replication of intracellular material. Alterations in cell cycle-related proteins have been well documented to disrupt normal cell division, ultimately contributing to cancer development [13]. Consequently, inhibiting the abnormal expression of these proteins can halt tumor cell growth and proliferation. Celastrol has been shown to inhibit proliferation by blocking C6 cells in the G_2_/M phase through the upregulation of the Bcl-2-associated X (Bax) and cleaved cysteinyl aspartate-specific proteinase 3 (caspase-3), and the related proteins cyclin B1 and the cyclin-dependent kinase inhibitors p21 and p27 [14]. In human gastric cancer BGC-823 and MGC-803 cells, celastrol inhibited p27 protein degradation by downregulating microRNA-21 (miR-21), thereby inducing G2/M cell cycle arrest [15]. Furthermore, Peng et al. [16] demonstrated that celastrol induced G0/G1 phase arrest in the human monocytic leukemia cell line U937 by reducing the levels of cell cycle proteins Cyclin D1, Cdk2, Cdk4, and Cdk6. Furthermore, tumor cell dormancy induced by cell cycle arrest is a key factor in tumor recurrence and distant metastasis after treatment, whereas transient cell cycle arrest allows tumor cells to repair genomic damage and maintain survival. This mechanism is of considerable significance and cannot be ignored in subsequent antitumor studies of celastrol.

#### 2.1.2. Inhibition of Tumor Cell Proliferation

Several studies have shown that celastrol inhibits the proliferation of a wide range of human tumor cells, including multiple myeloma (RPMI-8226), hepatocellular carcinoma (HepG2, C3A, and Huh7), gastric carcinoma (MKN45, MKN28, and KATOIII), prostate carcinoma (PC3, DU145, and LNCaP), renal carcinoma (RCC4), head and neck cancer (SCC4 and SCC38), non-small cell lung cancer (A549 and H1299), melanoma (SKMEL-28), neuroglioma (U251MG and U373MG), and breast cancer (MCF-7 and MDA-MB-231) [17]. Zhang et al. [18] demonstrated using a mouse model that celastrol inhibited the proliferation of human colon cancer cells (W620, RKO, and MC-38) in vivo. The study showed a significant reduction in tumor volume in the experimental group compared with the control group, further validating celastrol’s anti-proliferative activity. In a similar experiment, hepatocellular carcinoma cells (Hep3B) were implanted subcutaneously into athymic nude mice and treated with varying concentrations of celastrol. The results indicated that high doses of celastrol markedly suppressed Hep3B tumor growth, achieving a tumor growth inhibition rate of up to 52.9% [19].

#### 2.1.3. Mechanisms of Conventional Cell Apoptosis

Celastrol has the capacity to induce conventional apoptosis in various tumor cells through the intrinsic (mitochondrial), extrinsic (death receptor), and endoplasmic reticulum apoptotic pathways. It can regulate mitochondrial function via multiple targets, thereby activating the mitochondrial apoptotic pathway and exerting pro-apoptotic effects across different tumor types. Numerous studies have confirmed that celastrol induces apoptosis in melanoma, triple-negative breast cancer, and glioblastoma by reducing mitochondrial membrane potential and triggering mitochondrial dysfunction. This process leads to elevated levels of reactive oxygen species (ROS) and mitochondrial oxidative stress [20,21,22]. Moreover, celastrol upregulates the expression of pro-apoptotic proteins Bax and cytochrome c (Cyt c), downregulates the anti-apoptotic protein Bcl-2, and modulates the Bax/Bcl-2 ratio. It also increases cleaved caspase-3, -8, and -9, promotes the cleavage of poly (ADP-ribose) polymerase (PARP), and thereby activates the mitochondrial apoptotic pathway [23,24,25].

The extrinsic apoptotic pathway triggered by celastrol is primarily mediated by death receptors. Studies have shown that celastrol induces apoptosis in vincristine-resistant oral cancer cells by targeting the JNK1/2 signaling pathway [26]. In addition, by upregulating DR5 expression, celastrol promotes the binding of TNF-related apoptosis-inducing ligand (TRAIL) to death receptors, significantly enhancing its apoptosis-inducing effect in human glioblastoma cells [27].

The endoplasmic reticulum (ER) is the organelle responsible for protein synthesis and secretion. Under sustained or intense stress, endoplasmic reticulum stress (ERS) is activated. ERS can cause the accumulation of misfolded and unfolded proteins, triggering the unfolded protein response and initiating apoptotic cell death [28]. Ren et al. [29] demonstrated that celastrol induced apoptosis in human hepatocellular carcinoma cells (HepG2 and Bel-7402) by targeting ERS. ERS is often accompanied by the production of ROS, and excessive ROS can disrupt the cellular redox balance, thereby inducing apoptosis in NSCLC cells [30]. Similarly, treatment of human breast cancer MCF-7 cells with celastrol was found to induce apoptosis through activation of the ROS-protein kinase pathway, which involves the p53/polo-like kinase 2 (PLK2) signaling pathway [31].

#### 2.1.4. The Dark Side of Apoptosis

It is important to note that, although apoptosis is generally regarded as an ideal mechanism for antitumor therapy, several studies have suggested that inducing apoptosis in solid tumors may carry potential risks, and incomplete apoptosis can promote tumor repopulation [32]. Recent studies have shown that radiotherapy activates caspase-3, which subsequently cleaves calcium-independent phospholipase A_2_ (iPLA_2_). This cleavage leads to the release of arachidonic acid and its metabolism by COX-1/2 into prostaglandin E_2_ (PGE_2_). PGE_2_ is secreted by apoptotic cells into the tumor microenvironment, thereby promoting the proliferation of neighboring tumor cells [33]. Similar to the mechanism by which caspase-3 promotes tumor cell proliferation, tumor cells that enter the apoptotic program after chemotherapy can generate aneuploid and more invasive subpopulations at the late stages of apoptosis. In addition, defective apoptosis and apoptosis-induced nuclear extrusion may both contribute significantly to tumor drug resistance, recurrence, and metastasis [34,35]. These dark sides of apoptosis provide critical theoretical support for optimizing therapeutic regimens, avoiding adverse effects, and shifting antitumor strategies from solely promoting apoptosis to selectively inhibiting apoptosis in celastrol research.

Under pro-apoptotic conditions such as radiotherapy and chemotherapy, many tumor cells do not undergo complete cell death but instead form a specific type of highly malignant polyploid giant cancer cells (PGCCs), allowing tumor cells to enter a reversible state at the late stage of apoptosis [36]. PGCCs represent an important source of intratumoral heterogeneity. Although historically overlooked in studies of cell death, these cells exhibit exceptionally strong survival and proliferative potential. The presence of PGCCs has been documented in a wide range of solid tumors [37]. PGCCs can generate mononuclear progeny with stem cell-like properties through atypical mitotic division, such as neosis. This process produces tumor cell populations with distinct phenotypes, genotypes, and functions, thereby further amplifying and maintaining intratumoral heterogeneity. In addition, a subset of PGCCs can express immune cell-associated surface markers, including macrophage-related proteins, and these cells can migrate across blood vessels, promoting tumor metastasis [38]. This helps explain why therapeutic strategies that rely solely on apoptosis often fail to achieve durable antitumor effects. For the first time, Liu’s group definitively demonstrated that the atypical mitotic program employed by PGCCs can be pharmacologically inhibited [39]. Based on current evidence, we hypothesize that celastrol possesses multiple advantages in regulating cell division and interfering with signaling networks. Accordingly, it may represent a promising natural candidate for targeting PGCCs and suppressing their amitosis.

#### 2.1.5. Novel Regulatory Mechanisms of Specialized Cell Death Pathways

The dark side of apoptosis has driven a partial paradigm shift in tumor cell death research to some extent, encouraging investigators to focus on non-apoptotic forms of cell death. Unlike apoptosis, which carries a risk of inducing tumor repopulation, celastrol effectively triggers tumor cell death via novel regulated cell death pathways, including ferroptosis, cuproptosis and pyroptosis. The transition from classical apoptosis to these novel cell death modalities expands the cell death regulatory network and provides new strategies for clarifying disease pathogenesis and progression, as well as for identifying innovative therapeutic intervention targets.

Celastrol induces ferroptosis in tumor cells through a multitarget, multipathway mechanism, exhibiting distinct regulatory patterns across various cancer types, including gastric, hepatic, and pancreatic carcinomas. In gastric carcinoma, celastrol induces ferroptosis in HGC-27 cells by downregulating the key factor CERKL, which in turn suppresses the expression of ferroptosis marker proteins GPX4 and SLC7A11. Simultaneously, it promotes intracellular iron accumulation and ROS production, reduces glutathione (GSH) levels, and increases malondialdehyde levels. This oxidative stress imbalance ultimately leads to ferroptosis [40]. In hepatocellular carcinoma (HCC), the mechanisms are diverse. Celastrol can bind directly to cysteine residues of voltage-dependent anion channel 2 (VDAC2), triggering ROS-mediated ferroptosis and disrupting mitochondrial permeability transition pore function [41]. Alternatively, it can target RRM2 and suppress its expression, thereby downregulating mTOR phosphorylation and activating the ferroptosis pathway [42]. Using network pharmacology, Cai et al. [43] identified potential therapeutic targets in HCC and demonstrated that celastrol can induce ferroptosis by regulating GSTM1 expression. Furthermore, Yan et al. [44] found that celastrol triggers both autophagy and ferroptosis in SNU-423 cells by targeting the ferroptosis inhibitor FANCD2. In pancreatic cancer, celastrol binds directly to the Cys194 residue of sorcin, disrupting its interaction with PAX5. This promotes the nuclear translocation of PAX5 and reduces FBXL12 expression, which in turn decreases ALDH1A1 ubiquitination and induces ferroptosis in pancreatic cancer cells [45]. Collectively, these studies demonstrate that celastrol can induce ferroptosis in tumor cells through multiple pathways, including the regulation of iron homeostasis, control of oxidative stress, and modulation of signal transduction. This is achieved by targeting specific regulatory factors, such as CERKL, RRM2, FANCD2, and sorcin, as well as functional proteins like VDAC2. These findings provide a solid scientific basis for the development of celastrol as a ferroptosis-mediated anticancer agent.

Cuproptosis, a recently identified form of cell death, is primarily triggered by copper ion overload. Recent work by Xue et al. demonstrated that celastrol can upregulate the copper transporter SLC31A1, thereby promoting intracellular copper accumulation and reducing GSH levels. This leads to key hallmarks of cuproptosis, such as the loss of iron-sulfur clusters. Celastrol was also found to modulate ROS levels, mitochondrial membrane potential, and ATP levels. In vivo experiments showed that celastrol significantly inhibited tumor growth in mouse models without causing obvious cardiotoxicity, hepatotoxicity, or nephrotoxicity, supporting its potential as a safe and effective chemotherapeutic agent for non-small cell lung cancer [46].

**Figure 3:**
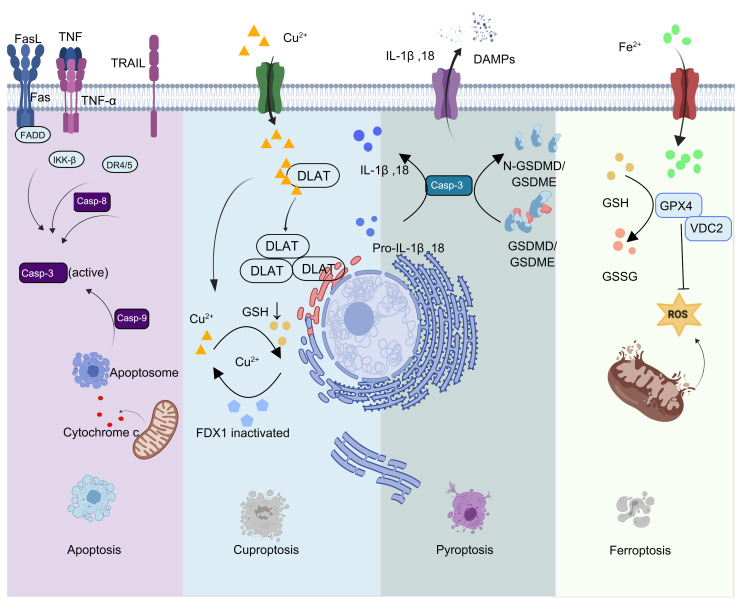
Four distinct types of programmed cell death mechanisms. Abbreviations: FasL, Fas Ligand; IKK-β, Inhibitor of Nuclear Factor Kappa B Kinase-beta; DLAT, Dihydrolipoamide S-acetyltransferase; DAMPs, Damage-Associated Molecular Patterns; GSDMD, Gasdermin D; GSDME, Gasdermin E; N-GSDMD, N-terminal Gasdermin D; N-GSDME, N-terminal Gasdermin E; GPX4, Glutathione Peroxidase 4; GSSG, Glutathione (oxidized form). Created with MedPeer (medpeer.cn).

Pyroptosis is a form of programmed cell death dependent on inflammatory caspases and is characterized by strong inflammatory responses. Guo et al. [47] first demonstrated that celastrol exerts anti-tumor effects by inducing ERS in tumor cells, thereby activating the caspase-3/GSDME-dependent pyroptosis pathway. This finding provides new experimental evidence for elucidating the molecular mechanisms underlying the multipathway tumor cell death induced by celastrol. Four types of programmed cell death, including apoptosis, cuproptosis, ferroptosis and pyroptosis, are shown in Figure 3.

Existing research has confirmed that celastrol is an effective inducer of immunogenic cell death (ICD) and can elicit antitumor immune responses in various tumor cell lines through distinct mechanisms. Wang et al. [48] successfully synthesized an ER -targeting celastrol nanoparticle that specifically induces ER stress in melanoma cells, thereby enhancing ICD. In colorectal cancer, celastrol triggers ICD by promoting ER stress and autophagy. Experiments using a KDEL peptide-modified extracellular vesicle (KME) delivery system to deliver celastrol along with programmed death ligand 1 (PD-L1) small interfering RNA (siRNA) to the ER showed that KME markedly amplifies celastrol-induced ICD. At the same time, it reduces the expression of both intracellular and membrane-bound PD-L1, promotes CD8^+^ T cell proliferation, and ultimately elicits potent antitumor immune responses [49]. Similarly, in clear cell renal cell carcinoma, celastrol induces ICD in 786-O cells by triggering autophagy and up-regulating the expression of high-mobility group box 1 and calreticulin, while simultaneously down-regulating PD-L1 through dual mechanisms: autophagy activation and MAPK1 inhibition [50]. These studies demonstrate that celastrol can activate antitumor immunity by inducing ICD and regulating PD-L1 expression, providing novel insights for combination immunotherapy across multiple malignancies.

#### 2.1.6. Induction of Cellular Autophagy

Autophagy is a double-edged sword in tumor progression. Moderate autophagy maintains cellular homeostasis and exerts antitumor activity, while aberrant or excessive autophagy promotes tumor progression and mediates therapy resistance [51]. Accumulating evidence indicates that celastrol regulates the initiation and execution of autophagy by regulating multiple key signaling molecules, including HSP90, MAPK, Beclin-1, ROS, NF-κB, AKT/mTOR, and the proteasome [52]. Additionally, numerous studies have shown that celastrol also induces autophagy and enhances apoptosis in other cancer cell lines. For example, Guo et al. [53] reported that celastrol downregulates the androgen receptor and its target miR-17-92a, leading to autophagy in prostate cancer cells; Li et al. [54] demonstrated that celastrol induces autophagy in human osteosarcoma cells. Collectively, these studies indicate that although excessive autophagy promotes tumorigenesis, celastrol can induce protective or cytotoxic autophagy in tumor cells via multipathway regulation under appropriate doses, thereby exerting significant antitumor effects.

#### 2.1.7. Inhibition of Tumor Cell Metastasis, Invasion and Adhesion

Metastasis refers to the process by which malignant tumors spread from their original site to other parts of the body, and is the leading cause of most cancer-related deaths worldwide [55]. For solid tumors, 66.7% of cancer deaths were registered with metastases as a contributing cause [56]. Invasion is a prerequisite of metastasis, and adhesion is a key regulator of metastasis [57]. Consequently, inhibiting tumor metastasis, invasion, and adhesion of malignant tumors is imperative. In vitro studies have demonstrated that celastrol exerts antitumor effects by inhibiting the gene products regulated by nuclear factor κB (NF-κB), thereby blocking tumor cell metastasis pathways [58]. Du et al. [59] reported that celastrol regulates metastasis in hepatocellular carcinoma cell lines (MHCC97H, Huh7, and HepG2) by inhibiting Rho-associated kinase 2 (ROCK2)-mediated phosphorylation of the Thr567 site on the connexin Ezrin. Furthermore, celastrol has been shown to impede the invasion of human breast cancer cells (MDA-MB-231) by TNF-α stimulation [60]. Zhu et al. [61] demonstrated that celastrol blocked extracellular matrix adhesion in human lung cancer 95-D cells and mouse melanoma B16F10 cells by activating the p38 MAPK signaling pathway and downregulating the phosphorylation of focal adhesion kinase (FAK), thereby inhibiting cell metastasis and invasion. In a separate study, Yao et al. [62] demonstrated that celastrol inhibited the metastasis, invasion and adhesion of MKN45 cells. Similarly, another study confirmed that celastrol suppressed the proteolytic activity of MMP-9 in human breast cancer MCF-7 cells, thereby hindering metastasis and invasion and exerting antitumor effects [63]. Collectively, these findings indicate that celastrol can inhibit tumor cell adhesion, invasion, and metastasis across various cancers by suppressing the expression and activation of key molecules, including ROCK2, FAK, and MMP 9, thereby exhibiting favorable antitumor activity.

#### 2.1.8. Celastrol Targeting Cancer Stem Cells

Celastrol has been extensively documented in oncological studies to exert potent antitumor activity by targeting cancer stem cells (CSCs) in various tumor types, modulating their stemness phenotypes and related signaling cascades. Mechanistically, celastrol directly inhibits the enzymatic activity of Pin1, thereby suppressing the Akt, STAT3, and NF-κB signaling pathways that govern CSC stemness. Consequently, it markedly downregulates the expression of core stem cell markers including CD44, Nanog, Oct4, and Klf4, reduces the CD44/CD24 stem-like cell population, and attenuates the stemness of ovarian CSCs [64]. In triple-negative breast cancer, Ramamoorthy et al. reported that celastrol selectively targets the Notch1–HES1/HEY1 axis, represses the expression of ALDH1, DCLK1, and CD133, and impairs tumor spheroid formation, thereby effectively eradicating triple-negative breast CSCs [65]. In thyroid cancer models, celastrol similarly downregulates Oct4, Rex1, and CD15, and strongly mitigates EMT-driven acquisition of cancer stem-like properties, ultimately restraining tumor initiation and progression [66]. Notably, the Moreira laboratory has consistently demonstrated in multiple studies that celastrol, either as a monotherapy or in combination with resveratrol, elicits robust antitumor efficacy against metastatic colorectal cancer and CSC populations [67,68,69].

### 2.2. Intervention Effect on the Tumor Microenvironment

**Figure 4:**
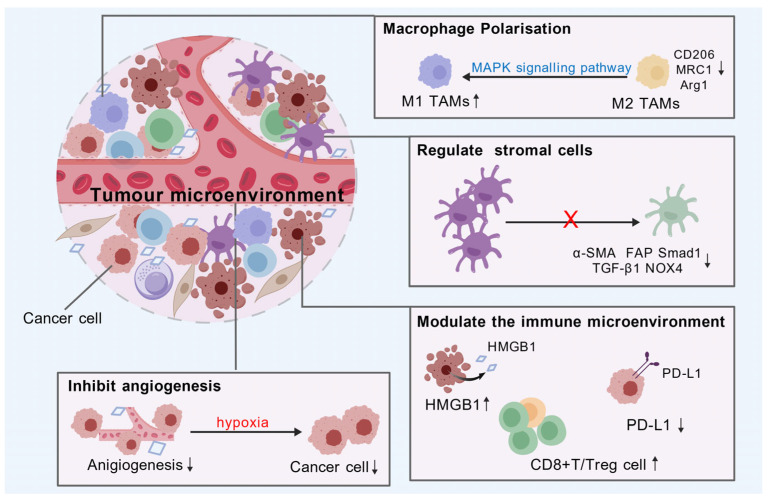
Celastrol’s regulatory mechanisms of the tumor microenvironment. Abbreviations: TAMs, tumor-associated macrophages; MRC1, mannose receptor 1; Arg1, arginase 1; α-SMA, alpha-smooth muscle actin; FAP, fibroblast activation protein; Smad1, SMA and MAD-related protein 1; TGF-β1, transforming growth factor-beta 1; NOX4, NADPH oxidase 4; HMGB1, high-mobility group box 1. Created with biogdp.com.

Tumor initiation, progression, and metastasis depend not only on the malignant proliferation of tumor cells themselves but also on the tumor microenvironment. Celastrol exerts significant antitumor effects primarily by remodeling the tumor microenvironment through multiple mechanisms, including inhibition of macrophage polarization, regulation of T cells, modulation of tumor-associated stromal cells, and suppression of angiogenesis. The regulatory mechanisms of the tumor microenvironment are illustrated in Figure 4.

One key mechanism by which celastrol exerts antitumor effects is through the regulation of macrophage polarization in the tumor microenvironment. In colorectal cancer, celastrol has been shown to repolarize macrophages from the M2 to the M1 phenotype via the MAPK signaling pathway in both in vitro and in vivo models, thereby remodeling the microenvironment to inhibit tumor growth [70]. In breast cancer, celastrol exerts a dose-dependent inhibitory effect on interleukin-13 (IL-13)-induced M2 macrophage polarization. This inhibition is accompanied by downregulation of M2-specific genes, including CD206, MRC1, and Arg1, and blockade of the key M2 polarization pathway through inhibition of STAT6 phosphorylation, resulting in a significant reduction of tumor metastasis in a mouse breast cancer model [71].

Of note, lactic acid and the acidic microenvironment generated by tumor glycolytic reprogramming play a crucial role in regulating the polarization of tumor-associated macrophages (TAMs) and immune cell infiltration, serving as key drivers in remodeling the tumor microenvironment. Studies have shown that celastrol suppresses glycolysis in intrahepatic cholangiocarcinoma cells, reduces lactate accumulation, and blocks tumor-promoting TAM polarization, thereby enabling dual regulation of tumor metabolism and the tumor microenvironment [72]. In hepatocellular carcinoma, celastrol indirectly inhibits glycolytic reprogramming by regulating the critical MBNL1-AS1/miR-708-5p/HK2 glycolytic axis, thereby overcoming resistance of HCC cells to celastrol [73].

The regulation of the T cell-associated immune microenvironment and the activation of antitumor immune responses are key pathways through which celastrol exerts its antitumor effects. In a mouse melanoma model, celastrol significantly inhibited tumor growth by increasing the number of CD8^+^ T cells, whereas it showed no antitumor activity in T cell-deficient nude mice. Moreover, combining low-dose celastrol with a TNFR2 antagonist synergistically increased the intratumoral CD8^+^ T cell/Treg ratio and suppressed Foxp3 expression in Treg cells, further enhancing T cell-mediated antitumor immunity [74]. In the treatment of triple-negative breast cancer, celastrol liposomes (Cel/Lip) inhibited cancer-associated adipocyte-mediated lipid infiltration, reduced the recruitment of immunosuppressive cells (e.g., MDSCs and Tregs), and enhanced the infiltration and activation of intratumoral CD8^+^ T cells, thereby remodeling the immunosuppressive tumor microenvironment [75]. Chen et al. [76] designed a hypoxia-responsive nanosystem (CS@TAP) for co-delivering celastrol and SN38, which synergistically induced ICD and remodeled the immunosuppressive microenvironment in colorectal cancer. In combination with an anti-PD-L1 monoclonal antibody, this nanosystem significantly enhanced immunogenic signals, promoted intratumoral CD8^+^ T cell infiltration, alleviated immunosuppression, inhibited tumor growth, and prolonged survival. Collectively, these findings indicate that celastrol remodels the immune microenvironment by enhancing effector CD8^+^ T cells, alleviating T cell exhaustion, and optimizing the ratio of intratumoral T cell subsets (CD8^+^ T cells/Tregs), thereby potentiating antitumor immunity.

Cancer-associated fibroblasts (CAFs) are the most abundant component of tumor stromal cells. CAFs strongly influence tumor growth and metastasis within the tumor microenvironment. A folate-modified multifunctional liposomal nano-delivery system co-loaded with celastrol and betulinic acid has been developed. In animal models of triple-negative breast cancer that simulate the tumor microenvironment, this nanoformulation effectively targeted CAFs, disrupted the tumor stromal barrier, and enhanced intratumoral drug accumulation, thereby significantly potentiating antitumor and antimetastatic efficacy [77]. This finding provides a novel therapeutic strategy by enabling celastrol to target both tumor cells and CAFs simultaneously. Therefore, effective tumor therapy may require not only direct targeting of tumor cells but also modulation of CAFs, which exert protumorigenic protective effects.

Celastrol also inhibits tumor growth by blocking angiogenesis and cutting off the nutrient supply to tumors. Neovascularization serves as a critical pathway for tumors to obtain nutrients and oxygen. In glioblastoma, angiogenesis and vasculogenic mimicry (VM) are essential for maintaining the tumor blood supply. Studies have confirmed that celastrol can significantly inhibit VM formation and angiogenesis. This is achieved by regulating the PI3K/AKT/mTOR signaling pathway, downregulating the expression of CD31, VEGFA, VEGFR2, EphA2, VE-cadherin, and HIF-1α, and suppressing glioma cell proliferation, migration, and invasion, thereby inhibiting tumor progression [78].

### 2.3. Multiple Signaling Pathways Working Together

Currently, the core signaling pathways involved in the regulation of celastrol’s antitumor activity, as reported in the literature, primarily include NF-κB, PI3K/AKT/mTOR, MAPK, STAT3 pathways, as well as apoptosis, invasion and migration, and autophagy-related pathways [79]. However, tumor cell suppression is rarely mediated by a single pathway; instead, multiple signaling pathways often act synergistically to exert antitumor effects. For example, in human multiple myeloma RPMI-8226 cells, celastrol inhibited tumor cell proliferation and induced apoptosis by activating the JNK pathway while inhibiting the PI3K signaling pathway. This resulted in reduced activation of caspase-3, -8, and -9, and suppressed the expression of the pro-apoptotic protein Bid and PARP cleavage [17]. Similarly, in ovarian cancer OVCAR3 cells [80] and human osteosarcoma U-2OS cells [81], celastrol negatively regulated tumor cell invasion and migration and induced apoptosis by downregulating mi-croRNA-21 and inhibiting the PI3K/AKT/NF-κB signaling pathway. These findings indicate that celastrol exerts broad-spectrum antitumor effects via multiple mechanisms, including inhibition of proliferation, induction of apoptosis, and suppression of invasion and migration. This highlights its multi-target, multi-pathway advantages in cancer therapy.

### 2.4. Summary of the Antitumor Mechanism of Celastrol

**Figure 5:**
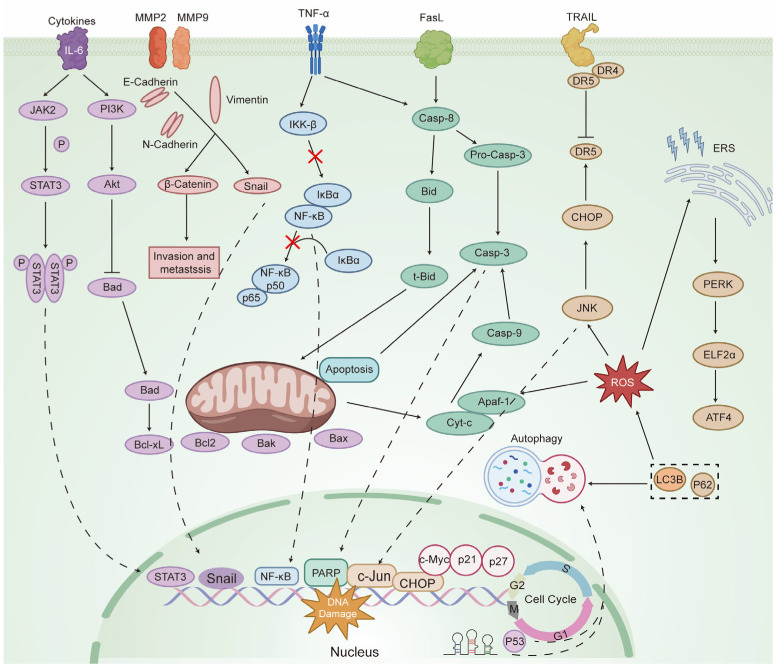
Antitumor mechanisms of celastrol. Celastrol exerts antitumor effects by regulating multiple signaling pathways. The red crosses indicate that celastrol inhibits the NF-κB pathway by blocking IκBα degradation. Celastrol suppresses tumor proliferation, invasion, and metastasis via inhibiting STAT3, PI3K/Akt, and EMT pathways; induces apoptosis, autophagy, and endoplasmic reticulum stress through death receptor, mitochondrial, and ROS-mediated pathways; and induces G2/M cell cycle arrest and DNA damage, ultimately leading to tumor cell death. Abbreviations: IL-6, interleukin-6; JAK2, Janus kinase 2; Bcl-xL, B-cell lymphoma-extra-large; IκBα, inhibitor of kappa B alpha; Bid, BH3-interacting domain death agonist; Apaf-1, apoptotic protease activating factor 1; c-Jun, Jun proto-oncogene; cyclin B1, G2/mitotic-specific cyclin-B1.

As discussed above, this article provides a comprehensive overview of the antitumor mechanisms of celastrol. Celastrol is a promising antitumor compound that targets multiple processes in tumor cells, including inhibition of migration and proliferation, as well as induction of autophagy and apoptosis. Subsequent studies on molecular mechanisms have revealed that these effects involve multiple signaling pathways. A summary of the molecular and cellular targets of celastrol is presented in Figure 5 and Table 1.

Celastrol exerts its biological effects via multiple mechanisms. It inhibits JAK2 kinase activity and prevents phosphorylation of STAT3 at Tyr705. Celastrol also blocks STAT3 dimerization and its nuclear translocation, thereby preventing it from binding to promoters of target genes such as c-Myc, Bcl-2, VEGF, and MMP-9. These actions collectively suppress tumor cell proliferation and induce apoptosis. In addition, celastrol inhibits the activation of AKT, a key downstream molecule in the PI3K pathway. Normally, AKT inactivates the pro-apoptotic protein BAD. When BAD is not phosphorylated, it binds to the anti-apoptotic protein Bcl-2, releasing Bax and Bak. This triggers changes in mitochondrial membrane permeability and the release of Cyt c, which activates the caspase cascade. Upon activation of caspase-3, PARP is cleaved, which inhibits DNA repair, induces DNA fragmentation, and ultimately leads to apoptosis. Celastrol also promotes apoptosis through the death receptor pathway. Caspase-8 acts as the initiator of extrinsic apoptosis by cleaving Bid to generate t-Bid, which transmits the death signal to mitochondria. This, in turn, activates the intrinsic apoptosis pathway, forming a central node where the extrinsic and intrinsic pathways intersect. Activation of the mitochondrial pathway disrupts the balance of the Bcl-2 family, leading to Bax/Bak oligomerization. This results in mitochondrial membrane permeabilization, Cyt c release, and apoptosome assembly via Apaf1. Subsequent activation of caspase-9 and caspase-3 completes the induction of apoptosis. Additionally, celastrol directly inhibits the IKK complex. It prevents IKKα phosphorylation, blocks its ubiquitination and degradation, stabilizes IKKα protein, and ultimately suppresses NF-κB nuclear translocation and DNA binding.

Celastrol can also promote mitochondrial fission by downregulating the expression of mitofusin-1, thereby disrupting the balance of mitochondrial dynamics. This disturbance leads to dysfunction of the mitochondrial respiratory chain and further drives the massive accumulation of ROS. Excessive ROS accumulation not only triggers the mitochondrial apoptotic pathway but also activates the p38 MAPK and JNK signaling pathways and upregulates the gene expression of the TRAIL death receptors DR4 (TRAIL-R1) and DR5 (TRAIL-R2). This facilitates the binding of additional TRAIL molecules to the receptor, thereby increasing the number of receptors available for recruitment. Consequently, more caspase-8 is recruited to the vicinity of the cell membrane, promoting its cleavage and inducing apoptosis. Furthermore, excessive accumulation of ROS leads to misfolding and aggregation of ERS-related proteins and the activation of the ERS transmembrane kinase PERK. PERK selectively promotes the translation of the transcription factor ATF4 by phosphorylating the translation initiation factor eIF2α. ATF4 then enters the nucleus and binds to the CHOP promoter, upregulating CHOP transcription and directly promoting cell apoptosis. Moreover, the process of ROS-induced autophagy has been observed to be a multifaceted phenomenon, mainly characterized by an increase in LC3B levels and a decrease in p62 levels.

The mechanism by which celastrol inhibits tumor cell migration and invasion is primarily associated with extracellular matrix (ECM) degradation and the epithelial–mesenchymal transition (EMT) process. Celastrol directly reduces the mRNA and protein levels of MMP-2 and MMP-9, thereby suppressing ECM enzymatic degradation and preventing tumor cells from penetrating the basement membrane. Twist, Slug, and Snail are key transcription factors that regulate EMT. They bind to the promoter region of E-cadherin, repress its transcription, and simultaneously upregulate mesenchymal markers such as N-cadherin and Vimentin, driving EMT progression. Celastrol reverses or inhibits EMT by downregulating Twist, Slug, and Snail, thereby blocking the activation of EMT signaling pathways upstream. This allows tumor cells to maintain an epithelial phenotype and reduces their migratory and invasive potential.

In summary, celastrol induces tumor cell apoptosis by targeting the STAT3, PI3K/AKT, and NF-κB pathways, as well as regulating ROS levels. These combined effects highlight its potential as a promising antitumor drug candidate.

## 3. Possible Reasons for the Potent Toxic Side Effects of Celastrol

The strong toxicity of celastrol is closely related to key modification sites and the activity of functional groups in its molecular structure. Its toxic effects mainly arise from the non-selective binding of highly reactive structural sites, the formation of toxic metabolites, and non-specific interactions between the molecule and cellular targets.

The carboxyl group at the C-20 position is a critical functional group that governs both the physicochemical properties and toxic accumulation of celastrol. Its high polarity results in extremely poor water solubility, causing the compound to accumulate in metabolic organs such as the liver and kidneys via passive diffusion. This accumulation exacerbates local tissue toxicity. Additionally, the carboxyl group can form hydrogen bonds or salt bridges with amino groups in normal cellular targets, increasing non-specific binding and broadening the scope of off-target damage. Structural modification of the C-20 carboxyl site can effectively reduce non-specific binding and toxicity while retaining, or even enhancing, the antitumor activity of celastrol [102].

The quinone methide moiety of Ring A may be one of the key structural groups responsible for celastrol’s toxicity. This quinone methide structure can form covalent bonds with nucleophilic groups, such as sulfhydryl and amino groups, in mitochondrial membrane proteins (e.g., VDAC1/2) and antioxidant enzymes (e.g., PRDX1) in normal cells, disrupting their normal functions. Such interactions directly impair cellular energy metabolism and ROS scavenging mechanisms, which can ultimately lead to cardiotoxicity, hepatotoxicity, neurotoxicity, and other adverse effects [103]. Furthermore, when the conjugated diene system of Rings A and B acts synergistically with the carbonyl group of Ring A, the carbonyl group enhances the electrophilicity of the diene system, while the planar structure of the diene stabilizes the binding conformation between the carbonyl group and target proteins [104]. These two moieties work together to impair protein function in normal tissues, thereby inducing toxicity.

The hydroxyl group at the C-3 position of celastrol exhibits a certain degree of nucleophilicity and can participate in oxidation reactions to generate toxic intermediates [105]. Moreover, celastrol may form covalent bonds with cytochrome P450 enzymes, impair hepatic metabolic function, and induce hepatotoxicity [106].

During the in vivo metabolic process of celastrol, some double bonds may be oxidized to generate toxic epoxide derivatives. According to the general mechanism of drug-induced toxicity, such reactive metabolites are highly electrophilic and readily form covalent bonds with DNA bases and protein side chains. This can lead to gene mutations and abnormal protein functions [107]. This may cause severe damage, especially to rapidly proliferating hematopoietic stem cells and germ cells, thereby inducing myelosuppression and reproductive toxicity.

## 4. Strategies to Improve the Activity and Reduce Toxicity of Celastrol

### 4.1. Antitumor Protein Targets of Celastrol

Celastrol is a typical natural small molecule containing a Michael acceptor moiety. It first engages with target proteins via specific noncovalent interactions, which precisely localize the molecule in proximity to reactive cysteine thiol groups, followed by the formation of reversible covalent bonds [108]. Through this dual binding mode combining noncovalent anchoring and reversible covalent conjugation, celastrol can directly interact with multiple intracellular proteins implicated in tumor progression. Accumulating evidence has confirmed several direct antitumor targets of celastrol, including Hsp90 co-chaperones, Nur77, PRDX1, and STAT3 [11]. Notably, CIP2A has also been definitively identified as a direct binding partner of celastrol. Upon binding, celastrol facilitates the interaction between CIP2A and CHIP, thereby mediating the ubiquitin-dependent degradation of CIP2A and suppressing the downstream Akt signaling pathway [109]. Furthermore, recent studies have further uncovered that VDAC2 is an additional direct binding target of celastrol. Specifically, celastrol directly binds to cysteine residues of VDAC2 and induces cyt-c release by dysregulating the function of VDAC2-mediated mitochondrial permeability transition pore, thereby exerting a potent anti-hepatocellular carcinoma effect [41].

### 4.2. Technologies for Target Identification of Celastrol

Target identification is the prerequisite and foundation for designing low-toxicity and high-efficacy derivatives. In recent years, researchers have established a variety of methods for identifying the pharmacological targets of natural products, such as activity-based protein profiling (ABPP) and derivative-based protein profiling (DBPP). Extending these strategies to the screening and identification of toxicological targets can provide important support for elucidating the mechanisms underlying drug-induced toxicity.

#### 4.2.1. Probe-Based Chemical Proteomics Technology

Chemical proteomics integrates chemical tools, such as small-molecule probes and bioactive compounds, with proteomics technologies to analyze protein functions, interactions, drug targets, and mechanisms of action. Traditional ABPP is the most established technique within chemical proteomics for identifying antitumor targets. This approach relies on the specific covalent binding of small-molecule probes to directly and selectively recognize target proteins.

ABPP has achieved significant breakthroughs in identifying drug efficacy targets. For example, Shi et al. [8] used ABPP to identify mitochondrial isocitrate dehydrogenases (IDH2 and IDH3A) as direct antitumor targets of celastrol in breast cancer. This study clarified the core mechanism by which celastrol inhibits mitochondrial metabolism and induces ferroptosis in tumor cells, providing critical target evidence to support its antitumor application.

ABPP technology also has valuable utility in identifying toxicological targets. We propose that applying ABPP probes to normal hepatocytes to specifically capture ferroptosis-related proteins or metabolic enzymes, such as members of the CYP450 family, that are covalently bound to celastrol, followed by validation of whether these proteins mediate celastrol-induced hepatotoxicity, could provide a solid foundation for elucidating its hepatotoxic mechanisms.

Although ABPP enables accurate target identification and direct validation of protein-small molecule interactions and binding sites, it relies heavily on rational probe design. It has limited capacity to detect low-abundance proteins and weak-interaction targets, and it cannot identify multiple targets simultaneously. Therefore, ABPP alone is insufficient to fully characterize the multi-target and multi-toxicity profiles of celastrol.

#### 4.2.2. Probe-Independent Proteomics Technology

To overcome the limitations of conventional ABPP, probe-independent proteomic technologies have emerged in recent years. A key advantage of these approaches is that they eliminate the need for small-molecule probe design. By integrating advanced technological strategies, they enable efficient capture of multiple and low-abundance targets, making them particularly suitable for identifying the toxicological targets of celastrol, including those associated with cardiotoxicity, reproductive toxicity, and neurotoxicity. Currently, these technologies primarily consist of two core methods.

The DBPP strategy, developed by Rao’s team, integrates proteolysis-targeting chimera (PROTAC) technology, quantitative proteomics, and immunoprecipitation-mass spectrometry (IP-MS). By establishing a degrader tool library, this approach enables the simultaneous identification of both known and novel targets. It is highly efficient, cost-effective, and probe-independent, while also capable of capturing multiple targets concurrently. This overcomes the limitations of traditional ABPP in multi-target identification. In identifying pharmacodynamic targets, the DBPP strategy has successfully revealed known antitumor targets of celastrol, such as PI3Kα and IKKβ, while simultaneously discovering novel antitumor-related targets, including CHK1 and OGA [110]. When applied to normal cardiomyocytes to screen for targets associated with cardiac function, the DBPP strategy is expected to efficiently identify unreported cardiotoxicity targets, thereby providing additional potential sites for the structural modification of celastrol to mitigate its toxicity.

Moreover, integrating the cellular thermal shift assay (CETSA) and pulse proteolysis [111] and thermostability-assisted limited proteolysis-coupled mass spectrometry (TALiP-MS) [112] has addressed challenges in identifying the targets of natural products, such as celastrol. These approaches overcome the limitations of traditional LiP-MS in detecting targets with minor structural changes or low abundance. CETSA-based strategies do not require probe design and are simpler to implement, providing a novel approach for identifying targets associated with reproductive toxicity or neurotoxicity.

Compared with ABPP, these probe-independent proteomic strategies are better suited for identifying the toxicological targets of celastrol. Nevertheless, ABPP remains unmatched in its precision for directly validating protein-small molecule interactions and characterizing binding sites.

#### 4.2.3. Network Pharmacology and AI-Assisted Technology

In recent years, network pharmacology has leveraged its strengths in multi-database integration and pathway analysis to efficiently identify potential toxicity targets of celastrol. Furthermore, the application of AI-assisted tools, including target prediction algorithms and molecular docking, can further improve the precision of target identification and clarify the underlying mechanisms of action. By integrating multi-source databases with computational simulations, these approaches enable rapid target screening and mechanistic prediction without requiring in vitro experiments. They complement the aforementioned proteomic technologies, further enhancing the accuracy and efficiency of celastrol target identification.

Xu et al. [113] developed a novel target enrichment and ranking tool called OTTER (online server: http://otter-simm.com/otter.html, accessed on 12 November 2025). Using literature mining and protein interaction analysis, they resolved the crystal structure of the celastrol-PRDX1 protein complex for the first time. It was shown that each PRDX1 dimer can covalently bind two celastrol molecules and additionally interact with adjacent dimers through hydrogen bonding and hydrophobic interactions. Such tight and irreversible binding may directly inactivate PRDX1, disrupt cellular redox homeostasis, and provide a crucial structural basis for celastrol’s toxic effects. Wang et al. [114] retrieved the key active components of *Tripterygium wilfordii Hook. f.* and screened for gene targets associated with ovarian toxicity via database searches. After identifying core targets through intersection analysis, molecular docking was performed to verify the binding affinity of the compounds to these targets. The results demonstrated that celastrol is a key compound inducing ovarian toxicity, with core toxic genes including TP53, MYC, PTEN, MAPK3, MTOR, STAT3, EGFR, KRAS, CDH1 and AKT1. Celastrol-induced apoptosis, dysregulation of signaling pathways, and other processes mediated by these genes may represent critical mechanisms underlying its ovarian toxicity, providing a core direction for future investigations into reproductive toxicity targets.

Currently, targets associated with reproductive toxicity and nephrotoxicity have been identified using methods such as ABPP and DBPP. These targets include HMGB1, VDAC1, PC, PKM2, FASN, and CHK1 [115]. Notably, many of these targets also contribute to antitumor activity. This dual role poses a core challenge in preserving the antitumor effects of celastrol while mitigating its toxicity.

We propose that future research on celastrol target identification should focus on two main directions. First, we need to further explore novel, specific toxicological targets and identify those that mediate toxicity without affecting the original antitumor activity. This approach would provide precise sites for selectively reducing toxicity. Second, we need to clarify the functional differences of targets that contribute to both antitumor activity and toxicity, and elucidate their regulatory mechanisms. These insights will provide a scientific basis for the structural modification of celastrol, ultimately achieving the goal of maintaining efficacy while minimizing toxicity.

### 4.3. Chemical Structural Modification of Celastrol Derivatives

Structural modification of natural products is a key strategy in drug development. Consequently, functional group modification of celastrol has become a major focus of research. To enhance antitumor activity and reduce toxicity, the carboxyl group at C-20 and the A/B rings of celastrol are commonly targeted for structural modification, providing abundant possibilities for derivatization. IC_50_ values obtained from porous plate assays reflect only the inhibition of cell proliferation, not the direct induction of cell death [116]. However, most derivatives are initially screened based on this index. Therefore, we systematically summarized and selected celastrol derivatives with excellent anti-proliferative activity from the literature and further analyzed their structure–activity relationships (SAR) in relation to both activity and toxicity, and also summarized the antitumor mechanisms of these derivatives in Table 2. This provides a scientific basis for the future molecular design and optimization of celastrol derivatives.

#### 4.3.1. Structural Modification of C-20 Position

**Figure 6:**
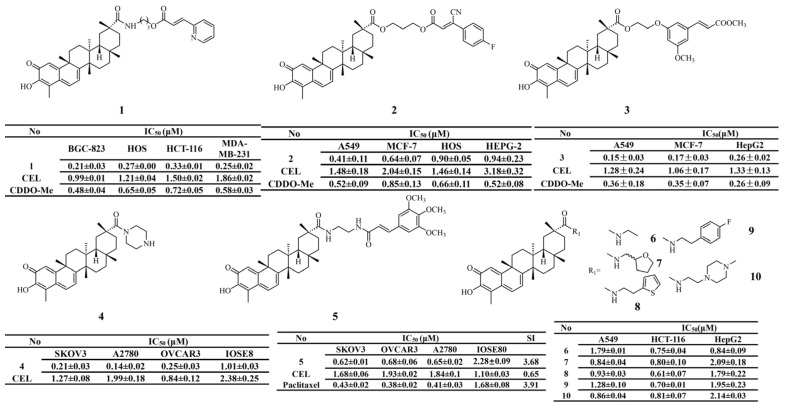
IC_50_ values of derivatives 1–10 in different tumor cells.

Numerous studies have shown that diverse modifications at the C-20 carboxyl group of celastrol yield distinct derivatives with inhibitory activity against various tumor cells. CK2 plays a critical role in the phosphorylation of Cdc37 at serine 13 during the Hsp90-Cdc37-kinase cycle. Zhang et al. [117] combined the pharmacophore of CK2 inhibitors with celastrol to enhance its antitumor activity. Derivative 1 (IC_50_ = 0.25 ± 0.02 μM) exhibited the most potent activity against MDA-MB-231 cells, showing approximately sevenfold higher potency than celastrol. Derivative 1 not only selectively inhibits CK2 activity but also disrupts the Hsp90-Cdc37 protein–protein interaction. In addition, derivative 1 displayed no obvious in vivo toxicity and achieved a high tumor inhibition rate of 65.3% (Figure 6). To develop novel and effective Hsp90-Cdc37 interaction-disrupting agents, Li et al. [118] introduced a series of lipophilic fragments at the C-20 position of celastrol and synthesized a panel of derivatives. The results revealed that derivative 2 (IC_50_ = 0.41 ± 0.11 μM) exhibited the most significant inhibitory effect on tumor cell growth, exceeding that of both celastrol and the control drug CDDO-Me. These studies demonstrate that structural modification at the C-20 position can significantly improve the in vitro antiproliferative activity of celastrol and enhance its ability to disrupt the Hsp90-Cdc37 interaction.

Introduction of bioactive functional fragments markedly enhances the antiproliferative activity of celastrol. A series of derivatives were generated by incorporating methyl ferulate fragments into celastrol using different linkers. Most of these derivatives exhibited stronger antiproliferative activity than celastrol. Notably, derivative 3 (IC_50_ = 0.15 ± 0.03 μM) showed the most potent inhibitory effect, with pharmacological activity comparable to or superior to that of the control drug [119]. Furthermore, in studies targeting ovarian cancer stem cells, derivatives 4 [120] and 5 [121] were obtained by directly introducing a piperazine moiety or a 3,4,5-trimethoxycinnamamide side chain at the C-20 position of celastrol. Both derivatives showed strong antiproliferative activity against SKOV3 ovarian cancer cells. Among them, derivative 5 (SI = 3.68) exhibited markedly higher selective cytotoxicity toward malignant cells than celastrol (SI = 0.65). The activity of derivative 4 depends on the inhibition of the STAT3 pathway, whereas no significant changes in this pathway were observed for derivative 5. In another study, an amide bond was introduced at the C-20 carboxyl group of celastrol. Derivatives 6–10 all displayed stronger antiproliferative activity than the parent compound. Among these derivatives, compound 8 (IC_50_ = 0.61 ± 0.07 μM) showed the strongest inhibitory activity across all tested tumor cell lines. Its antitumor activity was further evaluated in four human colorectal cancer organoid models. The results demonstrated that derivative 8 exhibited markedly superior activity compared with the positive control drug L-OHP in all models [122].

**Figure 7:**
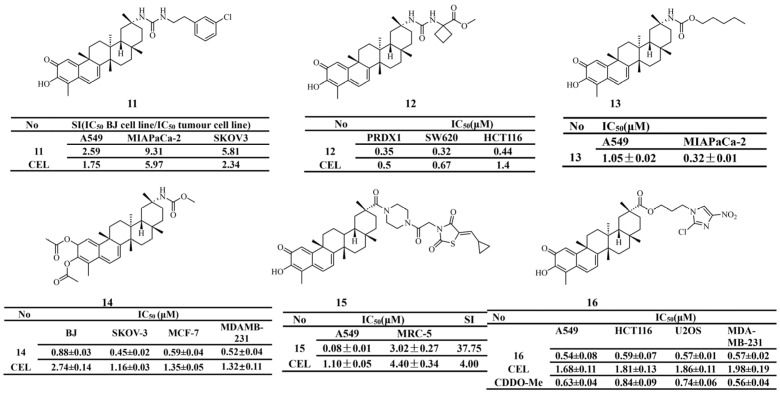
IC_50_ values of derivatives 11–16 in different tumor cells.

Introducing polar functional groups, such as urea and carbamate, or heterocyclic structures, such as thiazolidinedione, can improve selectivity toward tumor cells while reducing toxic side effects. Derivative 11, which contains a urea modification at the C-20 position, exhibits selective cytotoxicity against SKOV3 cells (IC_50_ = 0.56 μM, SI = 2.34) [123] (Figure 7). Derivative 12 (IC_50_ = 0.35 μM) shows potent anti-PRDX1 activity and strong antiproliferative effects against colon cancer cells. In a colorectal cancer xenograft model, it demonstrated significant antitumor efficacy with a more favorable safety profile than celastrol [124]. In a study by Sandra et al. [125], the C-20 carboxyl group was modified to generate a series of carbamate derivatives. Their antiproliferative activity was evaluated using MTT assays in A549 lung cancer cells and MIA PaCa-2 pancreatic cancer cells. The results showed that derivative 13 (IC_50_ = 0.32 ± 0.01 μM) had the most favorable biological activity. To further explore the potential of C-20 carbamate derivatives, selective acetylation of the A-ring followed by additional modification of the C-20 carboxyl group yielded derivative 14 (IC_50_ = 0.45 ± 0.02 μM). This compound exhibited antiproliferative activity against human ovarian cancer SKOV-3 cells. Moreover, combination treatment of derivative 14 with carboplatin produced synergistic antiproliferative effects in SKOV-3 cells, suggesting a promising strategy for ovarian cancer therapy. Derivative 15 was synthesized by introducing a thiazolidinedione moiety at the C-20 carboxyl group of celastrol using acetyl piperazine as a linker. It showed markedly enhanced inhibitory activity against non-small-cell lung cancer cells, with an IC_50_ value of 0.08 μM. This represents a 13.8-fold increase in potency compared with celastrol (IC_50_ = 1.10 μM). In a mouse xenograft model of A549 cells, derivative 15 effectively suppressed tumor growth [126].

**Figure 8:**
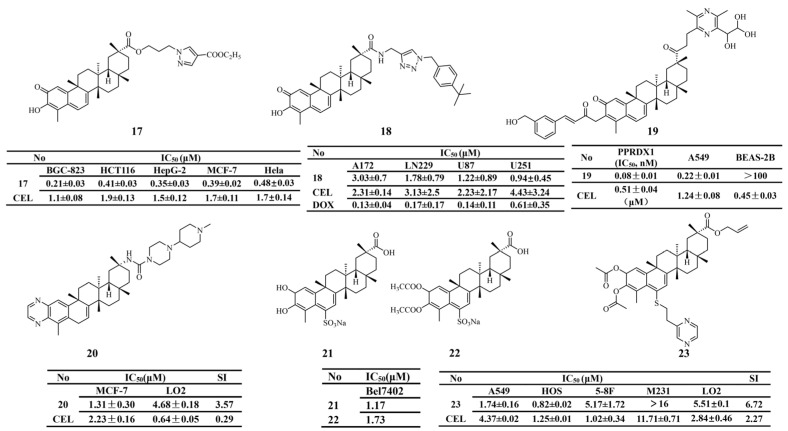
IC_50_ values of derivatives 17–23 in different tumor cells.

In recent years, azole compounds have been widely used in the structural modification of natural products. Li et al. enhanced the inhibitory effect of celastrol on the Hsp90-Cdc37 complex by introducing various imidazole substituents at the C-20 carboxyl group. Derivative 16, bearing chloro and nitro substituents, exhibited the strongest inhibitory activity against four tumor cell lines (A549, HCT116, U2OS, and MDA-MB-231) and effectively suppressed tumor growth in vivo [127]. Similarly, Lei et al. [128] introduced a pyrazole moiety at the C-20 carboxyl group. They found that derivative 17 (IC_50_ = 0.21 ± 0.03 μM) showed the most potent inhibitory effect on BGC-823 cells. Further studies demonstrated that derivative 17 exerted its antitumor activity by increasing ROS levels and inducing mitochondrial dysfunction in BGC-823 cells. In nude mouse xenograft models, derivative 17 achieved a tumor inhibition rate of up to 89.85% (Figure 8). In anti-glioma research, a celastrol-1,2,3-triazole derivative, 18, showed significant antiproliferative activity against U251 cells. It also markedly suppressed tumor cell proliferation in zebrafish xenograft models [129]. Collectively, these studies indicate that azole heterocycles are important moieties for C-20 structural modification of celastrol. The introduction of such heterocyclic substituents can significantly enhance antitumor activity. Moreover, derivatives containing different azole groups exhibit distinct mechanisms of action.

Furthermore, Guan et al. [130] identified five compounds targeting PRDX1 through a molecular docking-based screening approach. Among them, derivative 19 exhibited the most potent inhibitory activity (IC_50_ = 0.08 ± 0.01 nM) and showed high selectivity toward PRDX1. In addition, derivative 19 displayed no obvious hepatotoxicity or nephrotoxicity in mice. The chemical structures of derivatives 1–19 are illustrated in Figure 6, Figure 7 and Figure 8.

#### 4.3.2. Structural Modification of A/B Ring

Although relatively few structural modifications have been reported for the A/B rings of celastrol, such modifications can significantly influence its antitumor activity.

Using a skeleton transformation strategy, Feng et al. [131] introduced piperidine, pyrazine, and oxazole rings into the A ring of celastrol and incorporated a urea moiety at the C-20 carboxylate to evaluate antitumor activity. Derivative 20 (IC_50_ = 1.31 ± 0.30 μM, SI = 3.57), obtained by fusing a pyrazine ring onto the A ring and attaching a (1-methylpiperidin-4-yl) pyrazine moiety at the C-20 position, exhibited the strongest antiproliferative activity against MCF-7 cells in both in vitro and in vivo models, while showing reduced toxicity toward normal cells (Figure 8).

The characteristic quinone methide motif in the A/B rings of celastrol makes the C-6 position susceptible to nucleophilic attack. Based on this feature, Tang et al. [132] designed and synthesized C-6-substituted celastrol derivatives and evaluated their antiproliferative activity against human hepatocellular carcinoma Bel-7402 cells. The results showed that derivatives 21 (IC_50_ = 1.17 μM) and 22 (IC_50_ = 1.73 μM) exhibited strong antiproliferative activity against Bel-7402 cells in vitro. In vivo studies further indicated that derivative 22 was safer than derivative 21. In another study, Su et al. [133] reported that derivative 23 (IC_50_ = 0.82 ± 0.02 μM), which contains a pyrazine ring linked via a C-S bond, showed potent antiproliferative activity against human osteosarcoma HOS cells.

**Figure 9:**
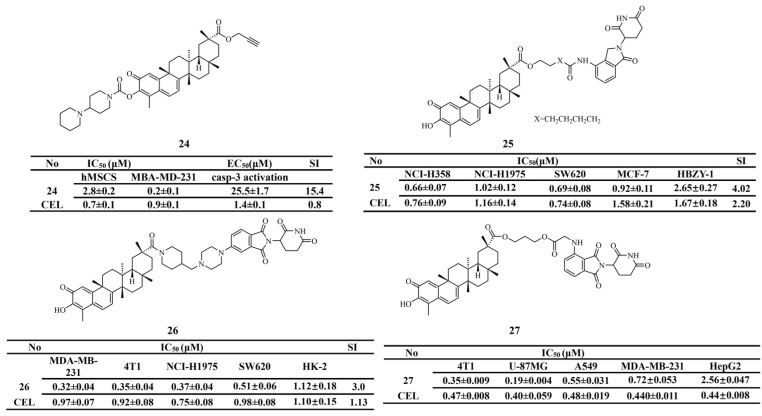
IC_50_ values of derivatives 24–27 in different tumor cells.

The hydroxyl group at the C-3 position of celastrol has been shown to interact with caspase-3, potentially leading to its overactivation. Hu et al. [134] modified the C-20 carboxyl moiety with a propargyl group and introduced a sterically bulky amide group at the C-3 position to synthesize derivative 24. This compound effectively inhibited the proliferation of MDA-MB-231 cells. It also showed markedly improved selectivity between human bone marrow-derived mesenchymal stem cells and MDA-MB-231 cells (SI = 15.4), compared with celastrol (SI = 0.8). Mechanistic studies demonstrated that derivative 24 exerted strong antitumor effects both in vitro and in vivo by disrupting the Hsp90-Cdc37 interaction and inhibiting angiogenesis. In addition, derivative 24 showed lower toxicity than celastrol and favorable in vivo pharmacokinetic properties (Figure 9). The structures of derivatives 20–24 are illustrated in Figure 8 and Figure 9.

#### 4.3.3. Design of PROTACTechnology-Mediated Derivatives

PROTACs are a widely studied technology that function by simultaneously binding to target proteins and E3 ligases, thereby inducing ubiquitination and subsequent proteasomal degradation [135]. Given the limitations of celastrol, this strategy has been increasingly applied to its structural design. In a study on non-small cell lung cancer, Ma et al. [136] developed nine celastrol derivatives using PROTAC technology. Among them, derivative 25 (IC_50_ = 0.66 ± 0.07 μM) showed the strongest inhibitory activity against NCI-H358 cells. In addition, derivative 25 exhibited 1.6-fold lower cytotoxicity toward normal cells than celastrol. Mechanistic studies demonstrated that derivative 25 effectively promoted the degradation of RAB9A, reduced AKT phosphorylation, and increased the expression of cleaved caspase-3.

Gu et al. [137] reported that novel derivative 26 (IC_50_ = 0.32 ± 0.04 μM) showed potent sensitivity toward MDA-MB-231 cells, with antiproliferative activity threefold higher than that of celastrol, while effectively reducing the expression levels of CDK4 and p-AKT. Subsequently, the researchers calculated the SI of derivative 26 for HK-2 and MDA-MB-231 cells. The SI value of derivative 26 (SI = 3.50) was significantly higher than that of celastrol (SI = 1.13), indicating improved tumor-selective cytotoxicity and reduced toxic side effects. Furthermore, through rational design, Gan et al. [138] developed derivative 27 (IC_50_ = 0.19 ± 0.004 μM). In vivo studies demonstrated that derivative 27 showed no obvious toxicity and effectively suppressed tumor growth. The structures of derivatives 25–27 are shown in Figure 9.

**Table 2:** Antitumor effects of celastrol derivatives on several malignant tumors.

Compd	Tumor Cell Lines	Main Mechanisms of Action	Ref
1	MDA-MB-231	Inhibit CK2 activity and disrupt the Hsp90-Cdc37 protein interaction.	[117]
2	A549, MCF-7, HOS and HepG2		[118]
3	A549, MCF-7 and HepG2	Destroys the Hsp90-Cdc37 protein complex, inhibits p-Akt and Cdk4 signaling, and induces tumor cell apoptosis via the death receptor pathway.	[119]
4	SKOV3 and A2780	Inhibits the STAT3 pathway and downregulates the expression of the stem cell markers CD44, CD24, and ALDH.	[120]
5	SKOV3 and OVCAR3	Reduces the proportions of CD44^+^, CD133^+^ and ALDH^+^, inhibits tumor cell proliferation, colony formation and sphere formation.	[121]
8	HCT-116	Binds to the SH2 domain of STAT3, inhibits its phosphorylation.	[122]
11	SKOV-3	Reduces p53 levels, inhibits Hsp90 protein, and modulates the Akt/mTOR signaling pathway.	[123]
12	SW620	Selectively inhibits PRDX1 activity, induces ROS accumulation and mitochondrial membrane potential depolarization.	[124]
14	SKOV-3	Inhibits the proliferation of tumor cells via the extrinsic apoptotic pathway and can be used in combination with carboplatin.	[125]
15	A549	Upregulates Bax expression, downregulates Bcl-2 expression, and activates the mitochondria-mediated apoptotic pathway.	[126]
16	A549, HCT116, U2OS and MDA-MB-231	Disrupt the Hsp90-Cdc37 protein interaction.	[127]
17	BGC-823	Increases ROS levels, induces mitochondrial damage, and activates the apoptotic pathway.	[128]
18	A172, LN229, U87 and U251	Activates the RIP1/RIP3/MLKL pathway and induces necroptosis in tumor cells.	[129]
19	A549, HepG2 and MCF-7	Binds to and inhibits PRDX1 activity.	[130]
20	MCF-7	Downregulates Cdk-1 and Cyclin B1, arrests the cell cycle at G_2_/M phase, and induces autophagy in tumor cells.	[131]
24	MDA-MB-231	Disrupts the Hsp90-CDC37 interaction and inhibits angiogenesis.	[134]
25	NCI-H358	Degrades RAB9A protein, inhibits the Akt pathway, and activates caspase-3-mediated apoptosis.	[136]
26	MDA-MB-231	Simultaneously degrades CHEK1 and PIK3R2, and reduces the levels of CDK4 and p-AKT.	[137]
27	4T1	Simultaneously degrades GRP94 and CDK1/4, inducing cell cycle arrest and apoptosis.	[138]

#### 4.3.4. SAR of Celastrol Derivatives

**Figure 10:**
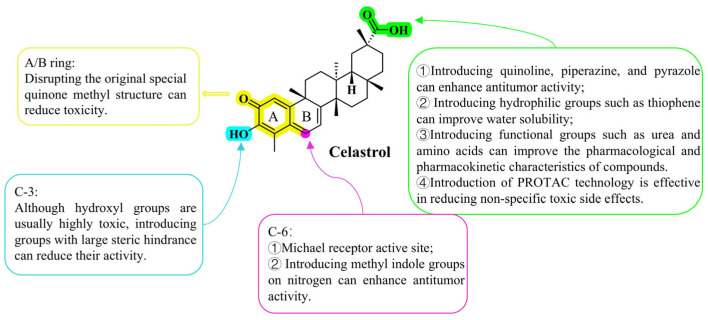
SAR of celastrol.

Despite the multiple modifiable sites present within the triterpene skeleton of celastrol, the natural product itself exhibits poor water solubility and high toxicity. Therefore, structural optimization guided by SAR studies is crucial to achieve a favorable balance between efficacy and toxicity. The synthetic strategies reviewed in this paper mainly include the following: (1) introduction of polar groups containing ester groups and amide bonds to improve antitumor activity; (2) introduction of groups with antitumor activity into the carboxyl group at the C-20 position of celastrol based on the principle of collocation of pharmacophore groups; (3) design of derivatives containing imidazole, pyrazole, and other structures at the C-20 position based on the principle of targeting action; (4) introduction of groups such as urea and amino acids into the C-20 position based on the principle of improving pharmacological activity and reducing the toxicity associated with the carboxyl group; (5) the unique quinone-methyl structure of the A/B ring makes the C-6 position a lively Michael addition receptor; (6) the introduction of a bulky group at the C-3 position has been demonstrated to reduce toxicity; (7) introduction of a pyrazine ring into the A ring disrupts the original quinone methyl structure, thereby reducing its toxicity; (8) application of PROTAC technology has enabled the precise reduction of its non-specific toxic side effects. The structure–activity relationship is shown in Figure 10.

### 4.4. Optimization of Nano Delivery Systems

Owing to their unique structures and properties, nano-delivery systems can effectively address key challenges associated with celastrol in antitumor applications, including high toxicity and low bioavailability [139]. Studies have confirmed that these systems represent a promising strategy to reduce toxicity and enhance the biological activity of celastrol. Nano-delivery systems can selectively target the tumor microenvironment and significantly increase drug accumulation in tumors. They also enable the synergistic co-delivery of multiple antitumor agents, thereby markedly enhancing the overall therapeutic efficacy of celastrol. This strategy has shown great potential in the treatment of various cancers, including ovarian [140], breast [141], pancreatic [142], liver [143], and oral squamous cell carcinoma [144]. Different delivery systems possess distinct features, although some common characteristics can be identified, as summarized below.

Nanocarriers can selectively recognize tumor cells or the tumor microenvironment. They promote drug accumulation at tumor sites, enhance targeting efficiency, and reduce toxic side effects. Peptide-modified cyclodextrin nano-delivery systems have been reported to enable the co-delivery of siPD-L1 and celastrol through hydrophobic and electrostatic interactions. This approach improves drug utilization and reduces adverse effects on normal tissues, thereby exerting potent antitumor activity [145]. Similarly, Yu et al. [146] used M1 macrophage-derived exosomes as nanoscale co-delivery vehicles. They constructed a delivery system by modifying it with the tLyP-1 targeting peptide and loading celastrol. The results showed improved targeting toward TNBC tumors, enhanced antitumor efficacy, reduced systemic toxicity, and better therapeutic outcomes. Encapsulation of celastrol in glycyrrhetinic acid (GA)-modified lipid calcium carbonate (LCC) nanoparticles significantly improved its targeting ability toward breast cancer [147]. In addition, glucose-functionalized mesoporous silica nanoparticles have been shown to be effective carriers for the targeted delivery of celastrol. This strategy not only improves water solubility but also selectively induces apoptosis in tumor cells [148]. Celastrol can also function as a multifunctional carrier for copper ions to form Cel-Cu nanoparticles (Cel-Cu NPs). These nanoparticles enable the controlled release of Cu^2+^ in the tumor microenvironment. The released Cu^2+^ binds to the acyl groups of the DLAT protein, thereby triggering cuproptosis. Meanwhile, celastrol further enhances this effect by inhibiting the NF-κB pathway, effectively reversing the immunosuppressive tumor microenvironment [149]. ROS-responsive polymeric micelles with specific affinity for hepatocellular carcinoma cells enable the combined use of celastrol and photodynamic therapy. This strategy addresses the issues of poor tumor targeting and low bioavailability, while further enhancing antitumor efficacy [150]. In addition, a multifunctional calcium carbonate-based nanoprobe (Fe_3_O_4_/CaCO_3_-CSL/ICG) integrates photo-therapy, chemotherapy, and ion interference therapy. It responds to the acidic tumor microenvironment and releases therapeutic agents in a controlled manner. This system enhances tumor suppression by inducing mitochondrial apoptosis through calcium overload and ROS accumulation [151]. Similarly, Zhang et al. [152] used an emulsification method to encapsulate CaCO_3_, mitoxantrone, and celastrol into pH-sensitive nanoparticles. These nanoparticles enable targeted release in the acidic tumor microenvironment. They trigger ICD and synergistically enhance antitumor immune responses. This nano-delivery system combines precise targeting with controlled release, thereby addressing key limitations in current research.

Some nanocarriers can achieve synergistic antitumor effects by co-delivering additional anticancer drugs, thereby enhancing therapeutic efficacy. In vitro studies have shown that composite nanoparticles co-loaded with axitinib and celastrol effectively inhibit angiogenesis, disrupt mitochondrial function, and suppress the proliferation of human breast cancer BT-474 cells, human neuroblastoma SH-SY5Y cells, and mouse squamous cell carcinoma SCC-7 cells. In a heterogeneous tumor model, mice treated with the composite nanoparticles exhibited a tumor inhibition rate of 64%, significantly higher than that of other groups [153]. Li et al. [154] developed a carrier-free, self-targeting nano-delivery system by fabricating celastrol-methotrexate (Ce-MTX) nanoparticles using the solvent precipitation method. Ce-MTX nanoparticles demonstrated stronger folate receptor-targeting ability than the free drugs. Moreover, in vivo studies confirmed that Ce-MTX effectively inhibited tumor growth while showing favorable biocompatibility. This nano-delivery system offers a novel and effective strategy for combination cancer therapy, with great potential for further development in nanotechnology and drug delivery.

The carrier materials used in current studies exhibit excellent biocompatibility, effectively reducing their own toxic side effects and enhancing the clinical translation potential of celastrol nanocarrier formulations. TiO_2_ nanofibers, recognized as highly biocompatible semiconductor nanomaterials, have been incorporated into celastrol delivery systems. Upon UV irradiation, the apoptosis rate of HepG2 cells increased to 43.9%, which was significantly higher than that of the control group [155]. Additionally, Li et al. [156] loaded celastrol into mesoporous polydopamine–polyethylene glycol (MPDA-PEG) nanospheres to construct the targeted delivery system MPDA-PEG-CLT. This system markedly enhanced targeted delivery efficiency and effectively induced apoptosis in osteosarcoma cells. Furthermore, studies have shown that using erythrocyte membranes—a biocompatible and readily available material—as a camouflage layer, combined with the cell penetrating peptide R8 to create a biomimetic nanoplatform for the co-delivery of triptolide and celastrol, enables precise induction of tumor cell apoptosis, suppresses tumor cell invasion, and disrupts autophagy in breast and liver cancers [157].

In addition, Li et al. [158] developed hybrid nanoparticles (Cel-TA-Cu NPs) by coordinating celastrol with Cu^2+^, followed by cross-linking with tannic acid (TA). This innovative nanosystem not only overcomes the inherent limitations of celastrol but also incorporates chemodynamic therapy, significantly enhancing therapeutic efficacy and offering a promising strategy for advanced tumor treatment.

Not only that, nanotechnologies such as supramolecular hydrogels [159], self-assembled nanoparticles [160,161], liposomes [162], micelles [163,164], biomimetic nanocrystals [165] and nanoemulsions [166] have been shown to improve bioavailability. Collectively, these nanomedical delivery systems hold great potential in cancer therapy due to their enhanced efficacy, improved water solubility, and increased bioavailability.

## 5. Conclusions and Outlook

As a natural product with broad-spectrum antitumor activity, celastrol offers the mechanistic advantage of regulating tumorigenesis and progression through multiple targets, providing a novel avenue for cancer therapy. Based on the preclinical evidence summarized in this review, celastrol and its derivatives show significant antitumor activity in various solid tumors. Among these, hepatocellular carcinoma, non-small-cell lung cancer, gastric cancer, breast cancer, and colorectal cancer may represent the most promising indications worthy of priority development. However, this multi-target property also contributes significantly to its severe toxicity. While modulating tumor-related pathways, celastrol can non-specifically bind to targets in normal cells, leading to mitochondrial dysfunction, oxidative stress imbalance, and aberrant signaling pathways. These effects ultimately manifest as multi-organ toxicities, including hepatotoxicity, cardiotoxicity, and hematotoxicity. Structural modifications at the C-20 carboxyl group and the A/B rings of celastrol allow for the synthesis of novel derivatives and the investigation of their structure–activity relationships. This approach facilitates the identification of intrinsic links between structure, activity, and toxicity, enabling the selection of derivatives with optimal antitumor activity, minimal toxicity, and high therapeutic potential. Nevertheless, efficient and precise screening of high-activity, low-toxicity derivatives still requires integration of advanced technologies. Currently, most structurally modified compounds remain at the laboratory stage, and clinical translation is a long-term goal. Similarly, the identification of toxicity targets is largely limited to mechanistic exploration, target screening, and methodology development, with substantial work still needed for clinical application. Nanomaterials, owing to their unique size, surface properties, and design flexibility, can reduce the toxicity of natural products through structural regulation and targeted delivery, providing a feasible strategy to overcome the intrinsic limitations of celastrol. Compared with other strategies, nanomedicine offers higher safety, favorable druggability, and a mature technical system, representing the most promising approach for clinical translation to achieve both toxicity reduction and efficacy enhancement. Nevertheless, the success of this strategy still depends on efficient preliminary derivative screening and in-depth mechanistic studies as its foundation. Therefore, it is currently possible to effectively mitigate the limitation of high toxicity through three primary strategies: combination strategy of technologies (toxicity target identification), structural modification (precision attenuation), and formulation optimization (targeted controlled release).

Research on the antitumor mechanisms of celastrol has progressed from single-pathway analyses to comprehensive studies of multi-dimensional network regulation. Future investigations should focus on the mechanisms by which celastrol exerts its effects through various forms of non-apoptotic regulated cell death, immunogenic cell death, and other pathways, as well as on its multi-pathway synergistic actions. Notably, while cell cycle arrest, apoptosis, and autophagy are key mechanisms mediating celastrol’s antitumor effects, their potential drawbacks should not be overlooked. Transient cell cycle arrest may allow tumor cells to repair genomic damage and enter a reversible dormant state, thereby evading therapeutic intervention. Similarly, dysregulated apoptosis and aberrantly activated autophagy can promote drug resistance, ultimately leading to tumor recurrence and treatment failure. These issues represent critical risks that warrant careful consideration in future studies of celastrol as an antitumor agent.

However, due to its severe toxicity, celastrol remains far from clinical application. In the future, integrating AI technologies will be essential for identifying the core targets responsible for celastrol-induced hepatotoxicity and cardiotoxicity, providing clear molecular guidance for precise toxicity-attenuation strategies. Based on the structural features of these toxic targets, targeted functional groups can be introduced into celastrol to block or weaken its interactions, achieving the dual goals of reducing toxicity while retaining pharmacodynamic activity. Combining nano-delivery systems with multi-modal therapies represents one of the most promising directions in antitumor research. This approach leverages precision delivery vehicles and synergistic treatment strategies to overcome key limitations of celastrol, including poor tumor targeting and significant side effects. Future development should focus on designing more precise targeting platforms that respond to the tumor microenvironment (e.g., pH- or enzyme-responsive mechanisms) to enable controlled drug release and selective targeting. Expanding synergistic therapeutic strategies, such as combining immunotherapy with radiotherapy, will further enhance antitumor efficacy while minimizing toxicity.

It is worth emphasizing that future research should continue to be driven by technological innovation and guided by clinical applications. Efforts will focus on deepening the understanding of the antitumor activity and mechanisms of celastrol and its derivatives, while integrating SAR analyses to provide new insights for the design and screening of novel celastrol derivatives.

## Figures and Tables

**Table 1:** Antitumor effects of celastrol on several malignant tumors.

Type of Cancer	Tumor Cell Line	Main Mechanisms of Action	In Vivo Antitumor	Ref
Colorectal cancer	HCT-116	inhibits Nur77 and induces high expression of ATG7 signaling.	Celastrol (1.25, 2.5 mg/kg, i.p. qod × 24 d), significantly suppressed tumor growth in nude mouse xenografts	[82]
	HCT-116 and SW620	reduces the expression of TGF-β1, TGFβRI and TGFβRII, and inhibits the upregulation of Smad4 and p-Smad2/3.	Not evaluated in vivo	[83]
Colorectal cancer	HT-29 and HCT-119	inhibits NOS activity and suppresses the angiogenic pathway.	Not evaluated in vivo	[84]
	SW480 and HCT116	affects the components of the PI3K/AKT pathway and the expression of MMP3 and MMP7.	Not evaluated in vivo	[85]
Prostate cancer	DU145	blocks the hERG channel.	Not evaluated in vivo	[86]
	PC-3 and LNCaP	inhibits the activities of 20S and 26S proteasomes.	celastrol (1–3 mg/kg/d), significantly suppressed tumor growth (65–93%) in PC-3 xenografts	[87]
Renal cell carcinoma	ACHN and Caki-1	upregulates TRAIL-R2 and activates the caspase cascade.	Not evaluated in vivo	[88]
Gastric cancer	MKN45	downregulates miR-21 and inhibits the PTEN/PI3K/AKT and NF-κB signaling pathways.	Not evaluated in vivo	[62]
	SGC-7901 and BGC-823	inhibits Prdx2 and induces intracellular ROS accumulation.	Celastrol (1.5 mg/kg) every other day for 24 days, significantly suppressed tumor growth in nude mouse xenografts	[9]
	BGC-823, SGC-7901 and MGC-803	upregulates the expression of miR-146a and inhibits NF-κB activity.	Not evaluated in vivo	[89]
	BGC-823 and MGC-803	inhibits the miR-21-mTOR signaling pathway and increases the level of p27.	Not evaluated in vivo	[15]
Multiple myeloma	CD138^+^	upregulates caspase-3, inhibits NF-κB activation and expression of CXCR4 and MMP-9, and reduces serum levels of IL-6 and TNF-α.	Celastrol (0.25 mg/kg, 5 d/week × 3 weeks), suppressed tumor growth in nude mouse xenografts	[90]
	LP-1 and RPMI 8226	inhibits the NF-κB pathway.	Not evaluated in vivo	[91]
Breast cancer	MDA-MB-231	inhibits TNF-α-induced MMP-9 gene expression.	Not evaluated in vivo	[60]
		inhibits the HSDL2/MAPK/ERK signaling pathway.	Not evaluated in vivo	[92]
	MCF-7	inhibits TNF-α-induced MMP-9 gene expression.	Not evaluated in vivo	[63]
		activates caspase-7, 8, 9, promotes PARP cleavage, and release of Cyt c and AIF, downregulates Bcl-2, upregulates Bax, and mediates mitochondrial apoptosis.	Not evaluated in vivo	[93]
Adenoma	AtT 20	downregulates AKT/mTOR signaling.	Celastrol (2 mg/kg × 14 d), significantly suppressed tumor growth in nude mouse xenografts	[94]
Non-small cell lung cancer	H460, PC-9 and H520	promotes ROS accumulation and inhibits the STAT3 pathway.	Celastrol (4 mg/kg, qod × 15 d), significantly suppressed tumor growth in nude mouse xenografts	[30]
	A549	activates the mitochondrial pathway and FasL-mediated pathway.	Not evaluated in vivo	[23]
	H1299, H226 and H522	regulates the circ-SATB2/ miR-33a-5p/E2F7 signaling cascade.	Celastrol (2 mg/kg, every 5 d × 4 doses), significantly suppressed tumor growth in nude mouse xenografts	[95]
Human esophageal cancer	Eca109 and ECI	activates ATF4 transcription and inhibits the FoxO3a-Bim pathway.	Celastrol (4, 8 mg/kg, i.p. qod × 18 d), suppressed tumor growth in nude mouse xenografts	[96]
Human osteosarcoma	U-2OS	induces apoptosis via the mitochondrial pathway.	Not evaluated in vivo	[24]
	HOS, MG-63, U-2OS and Saos-2	induces G_2_/M phase arrest, apoptosis and autophagy through the ROS/JNK signaling pathway.	Celastrol (1, 2 mg/kg), significantly reduced tumor volume by 42.9% and 50.2% after 7 days, respectively	[54]
	U-2OS	downregulates the PI3K/AKT/NF-κB signaling pathway.	Not evaluated in vivo	[81]
Ovarian cancer	SKOV-3 and OVCAR-3	downregulates miRNA-21 and the PI3K/AKT-NF-κB signaling pathway.	Not evaluated in vivo	[80]
		inhibits IκBα phosphorylation, prevents IκBα degradation, and blocks p65 nuclear accumulation, thereby disrupting the classical NF-κB pathway.	Not evaluated in vivo	[97]
Hepatocellular carcinoma	HepG2	downregulates the mRNA and protein expression of E2F1.	Not evaluated in vivo	[98]
	SK-Hep1	inhibits the mTOR/ERK pathway, reduces HIF-1α protein synthesis, and downregulates the expression of VEGF and EPO.	Celastrol (3, 10 mg/kg, p.o. 3 times/week × 35 d), significantly suppressed tumor growth in nude mouse xenografts	[19]
	MHCC97H	inhibits ROCK2-mediated phosphorylation of Ezrin at Thr567.	Not evaluated in vivo	[59]
	SNU-423 and SNU-387	targets FANCD2 to induce autophagy-dependent ferroptosis.	Not evaluated in vivo	[44]
Pancreatic cancer	PANC-1, SW1990, AsPC-1 and BxPC-3	induces apoptosis via m6A-YTHDF3-mediated downregulation of Claspin and Bcl-2.	Celastrol (1.0, 3.0 mg/kg, i.p. q2d × 35 d), significantly suppressed tumor growth in nude mouse xenografts	[99]
Melanoma	B16-F10	inhibits the PI3K/AKT/mTOR pathway and regulates the expression of downstream HIF-α mRNA.	Not evaluated in vivo	[20]
Thyroid cancer	MDA-T32 and KTC-1	promotes apoptosis via the caspase-3 pathway.	Celastrol (1, 2 mg/kg, i.p. qod × 14 d), significantly suppressed tumor growth in nude mouse xenografts	[100]
Medulloblastoma	SHH-MB	increases ROS levels, downregulates TGF-β2 and CXCL12 mRNA, and inhibits the NF-κB pathway.	Celastrol (2 mg/kg, i.p. qd × 20 d), significantly suppressed tumor growth in nude mouse xenografts	[101]

## Data Availability

No new data were generated or analyzed in this study.

## Abbreviations

The following abbreviations are used in this manuscript: ABPP, activity-based protein profiling; Apaf-1, apoptotic protease activating factor 1; AKT, Protein kinase B; α-SMA, alpha-smooth muscle actin; Arg1, arginase 1; Bax, Bcl-2-associated X; Bcl-xL, B-cell lymphoma-extra-large; Bid, BH3-interacting domain death agonist; CAFs, cancer-associated fibroblasts; caspase-3, cysteinyl aspartate-specific proteinase 3; CSCs, cancer stem cells; Ce-MTX, celastrol-methotrexate; CHOP, C/EBP homologous protein; c-Jun, Jun proto-oncogene; c-Myc, myelocytomatosis oncogene; cyclin B1, G2/mitotic-specific cyclin-B1; Cyt c, Cytochrome c; DBPP, Degradation-Based Protein Profiling; ERS, endoplasmic reticulum stress; FasL, Fas ligand; FAP, fibroblast activation protein; GA, glycyrrhetinic acid; GSH, glutathione; HMGB1, high-mobility group box 1; ICD, immunogenic cell death; IκBα, inhibitor of kappa B alpha; IKK-β, inhibitor of nuclear factor kappa-B kinase subunit beta; IL-6, interleukin-6; IL-13, interleukin-13; JAK2, Janus kinase 2; JNK, c-Jun N-terminal kinase; MRC1, mannose receptor 1; MPDA-PEG, mesoporous polydopamine–polyethylene glycol; miR-21, microRNA-21; MM, multiple myeloma; NF-κB, nuclear factor κB; NOX4, NADPH oxidase 4; PARP, poly ADP ribose polymerase; PD-L1, programmed death ligand 1; PGE_2_, prostaglandin E_2_; PGCCs, Polyploid Giant Cancer Cells; PROTAC, Proteolysis-Targeting Chimera; ROCK2, Rho-associated kinase 2; ROS, reactive oxygen species; siRNA, small interfering RNA; Smad1, SMA and MAD-related protein 1; STAT3, signal transducer and activator of transcription 3; TAMs, tumor-associated macrophages; TALiP-MS, thermostability-assisted limited proteolysis-coupled mass spectrometry; TGF-β1, transforming growth factor-beta 1; TNF, tumor necrosis factor; TRAIL, TNF-related apoptosis-inducing ligand; VDAC2, voltage-dependent anion channel 2; VEGF, vascular endothelial growth factor.
